# Association between renal function and platelet reactivity during aspirin therapy in elderly patients with atherosclerotic cardiovascular disease

**DOI:** 10.1186/s12877-021-02018-y

**Published:** 2021-01-22

**Authors:** Wenyi Liang, Peng Zhang, Meilin Liu

**Affiliations:** Department of Geriatrics, Peking University First Hospital, No. 8, Xishiku Street, Xicheng District, Beijing, 100034 People’s Republic of China

**Keywords:** Age, Aspirin, Atherosclerotic cardiovascular disease, Platelet reactivity, Renal function

## Abstract

**Background:**

Aspirin is the key treatment in the secondary prevention of atherosclerotic cardiovascular disease. High on-treatment platelet reactivity (HTPR) to aspirin has been reported to partially account for the enhanced risk of thrombotic events. In particular, HTPR has been described more frequently among elderly patients. The aim of this study was to identify the clinical and biological factors associated with HTPR in a real-life elderly population.

**Methods:**

In this retrospective study, elderly patients with atherosclerotic cardiovascular disease on regular aspirin treatment were enrolled. Cardiovascular risk factors, routine biological parameters, comorbidities, and concomitant medications were recorded. The upper quartile of the platelet aggregation rate, determined by light transmission aggregometry with arachidonic acid, was defined as the HTPR group.

**Results:**

A total of 304 patients were included (mean age 77 ± 8 years, 76% men). Patients in the HTPR group were older than the patients in the non-HTPR group (mean age: 79 ± 7 vs. 76 ± 8 years, *p* = 0.008). Patients with moderately decreased estimated glomerular filtration rate (eGFR) had a higher frequency of HTPR than patients with slightly decreased eGFR or normal eGFR (35.8, 22.5, 12.2%, respectively, *p* < 0.05). In multivariate analysis, an independent risk factor for HTPR was the eGFR (OR: 0.984, 95% CI: 0.980–0.988, *p* < 0.001).

**Conclusions:**

Advanced age and decreased eGFR are correlated with poor pharmacodynamic response to aspirin.

## Background

Atherosclerotic cardiovascular disease (ASCVD) includes coronary heart disease, ischemic stroke or transient ischemic attacks, and peripheral arterial disease, all of presumed atherosclerotic origin [1]. Aspirin is the key treatment in the secondary prevention of ASCVD due to its prominent antiplatelet effects [2, 3]. Aspirin impedes thrombus formation by inhibiting cyclooxygenase (COX)-1, which mediates thromboxane A2 (TXA2) synthesis [4, 5].

Recent studies have shown that aspirin fails to prevent a number of serious cardiovascular events among high-risk patients [6, 7]. This has led to the introduction of the concept of high on-treatment platelet reactivity (HTPR) [8]. In particular, HTPR has been described more frequently among elderly patients, conditioned by impaired drug absorption and metabolism and by a baseline of more enhanced platelet reactivity. Most previous studies enrolled patients receiving dual antiplatelet therapy [9, 10]. However, there is evidence of overlap in the antiplatelet effects of aspirin and P2Y12 antagonists [11]. The main objective of our study was to identify routinely available clinical and biological factors associated with HTPR in a real-life elderly population of ASCVD patients receiving aspirin as monotherapy. We hypothesized that finding factors associated with HTPR can help identify high-risk patients and eventually modify antiplatelet therapy.

## Methods

### Study design and participants

Elderly patients on regular aspirin treatment in the Department of Geriatrics of Peking University First Hospital were enrolled from March 2014 to December 2019.

The inclusion criteria were as follows:

1. Age: ≥ 60 years;
2. Diagnosis of ASCVD, presence of at least one of the following diseases: coronary heart disease, ischemic stroke, transient ischemic attacks, and peripheral arterial disease;
3. Aspirin monotherapy was used for secondary prevention (50–100 mg daily for more than 1 month) without a change in dose within the previous month. Compliance with aspirin was verified separately by the patient’s attending physician and study physician.

The exclusion criteria were as follows:

1. Use of other antithrombotic medications (including clopidogrel, ticagrelor, prasugrel, dipyridamole, warfarin, heparin, low-molecular-weight heparin and new oral anticoagulants) or nonsteroidal anti-inflammatory drugs within 1 month;
2. Severe renal dysfunction [estimated glomerular filtration rate (eGFR) < 30 ml/min/1.73 m^2^] or dialysis;
3. Platelet count < 100 ×  10^3^/μL or > 450 × 10^3^/μL;
4. A major surgical procedure or severe inflammation within 1 week;
5. History of malignant tumor, chronic inflammatory diseases, serious liver disease and autoimmune disease.

### Medical records collection

For each patient, detailed medical records were obtained from the electronic medical record system, including age, sex, smoking habit, alcohol habit, body mass index, comorbidities, concomitant medications, and usual biological parameters.

### Platelet reactivity

For each patient, platelet aggregation was determined by light transmission aggregometry with arachidonic acid (LTA-AA). Platelet aggregation tests have been routinely performed for several years at our clinical laboratory by experienced technicians. Blood samples were collected within 21–22 h after aspirin intake and analyzed within 1 h from collection. Blood samples anticoagulated with 3.2% sodium citrate were centrifuged at 200×*g* for 10 min to obtain platelet-rich plasma and further centrifuged at 2000×*g* for 10 min to obtain platelet-poor plasma. Platelet aggregation was performed by the addition of 0.5 mg/mL arachidonic acid to platelet-rich plasma cuvette using platelet-poor plasma as a reference cuvette. The percentage of platelet aggregation was defined as the maximal light transmittance after arachidonic acid addition measured by the LBY-NJ4 platelet aggregometer (PRECIL, Beijing, China) [12].

### Patient group

Patients were divided according to LTA-AA. Patients with platelet aggregation rates in quartile IV were defined as the HTPR group, whereas patients with platelet aggregation rates in quartiles I-III were defined as the non-HTPR group.

Patients were also stratified by eGFR, which was calculated using the MDRD (Modification of Diet in Renal Disease) formula [13]. The G1 group included patients with normal eGFR (eGFR ≥90 mL/min/1.73 m^2^). The G2 group included patients with slightly decreased eGFR (eGFR 60–89 mL/min/1.73 m^2^). The G3 group included patients with moderately decreased eGFR (eGFR 30–59 mL/min/1.73 m^2^).

### Statistical analysis

The distribution normality of each variable was tested using the Kolmogorov-Smirnov test. Continuous variables were expressed as the mean ± SD or median (interquartile range). Homogeneity of variance was tested for continuous variables. For normally distributed data, comparisons between two groups were performed by Student’s unpaired *t* test, and comparisons among groups were performed using one-way ANOVA, followed by post-hoc analysis of LSD tests (if variances were equal) or Tamhane’s T2 tests (if variances were unequal). The nonparametric Mann-Whitney U test or Kruskal-Wallis H test were performed if the data were nonnormal. Categorical variables were described as counts (percentages) and were tested using the chi-square test. Correction for multiple comparisons was performed using the Bonferroni-Holm correction. Correlations between quantitative variables were assessed using Pearson or Spearman correlation coefficients, as appropriate. Multivariate logistic regression analysis (forward: conditional) was performed to investigate risk factors for HTPR. After univariate analysis, variables that presented significant associations with HTPR (*p* < 0.10) were entered in the multivariate model. Differences were considered significant when *p* < 0.05 (two-tailed). The cutoff value was calculated by analyzing the receiver operating characteristic (ROC) curve. Statistical analysis was carried out using SPSS version 16.0 software (SPSS Inc., Chicago, IL, USA).

## Results

Finally, 304 patients with sufficient available clinical and biological information were selected for this study. The average age was 77 ± 8 years, and 233 (76.6%) patients were males. There were 120 (39.5%) patients with age ≥ 80 years and 8 (2.6%) patients with age ≥ 90 years.

### Testing of platelet aggregation

During aspirin therapy, the range of LTA-AA was from 0.84 to 34.91%, with a median value of 9.69% (IQR 6.80–12.38%). There were 3 (0.99%) patients with LTA-AA > 20%. According to the aforementioned definition, there were 76 patients in the HTPR group (LTA-AA ≥12.38%), with a median value of 14.41% (IQR 12.94–16.82%), and there were 228 patients in the non-HTPR group (LTA-AA < 12.38%), with a median value of 8.69% (IQR 5.83–10.48%).

### Clinical features of elderly patients with ASCVD classified by HTPR

The clinical and biological characteristics of the study participants divided according to HTPR are shown in Table 1 and Table 2. Patients in the HTPR group were older than patients in the non-HTPR group (mean age: 79 ± 7 vs. 76 ± 8 years, *p* = 0.008). In terms of the ASCVD spectrum, patients in the HTPR group had an increased presence of ischemic stroke or transient ischemic attacks, and fewer patients had a history of coronary heart disease than those in the non-HTPR group (*p* < 0.01). The ratio of angiotensin converting enzyme inhibitors/angiotensin receptor blockers (ACEIs/ARBs) medications was higher in the HTPR group (*p* = 0.046). There were no significant differences in daily aspirin dose between the two groups (*p* > 0.05). Patients in the HTPR group had lower levels of eGFR (average values: 65.2 vs. 71.8 mL/min/1.73 m^2^, *p* = 0.002), indicating poorer renal function than patients in the non-HTPR group. The hemoglobin level was significantly lower in the HTPR group than that in the non-HTPR group (*p* < 0.001).

**Table 1:** Clinical characteristics of study participants classified by HTPR

Variables	HTPR group (*N* = 76)	non-HTPR group (*N* = 228)	*p* value
Age, years	79 ± 7	76 ± 8	0.008
Male sex, *n* (%)	54 (71.1)	179 (78.5)	0.183
Body mass index, kg/m^2^	24.0 ± 2.7	24.6 ± 3.1	0.149
Systolic blood pressure, mmHg	138 ± 18	134 ± 16	0.076
Diastolic blood pressure, mmHg	71 ± 11	73 ± 11	0.164
Medical history			
Coronary heart disease, *n* (%)	59 (77.6)	210 (92.1)	0.001
PCI, *n* (%)	33 (43.4)	115 (50.4)	0.289
Ischemic stroke or TIA, *n* (%)	39 (51.3)	56 (24.6)	< 0.001
Peripheral artery stenosis, *n* (%)	7 (9.2)	29 (12.7)	0.412
Hypertension, *n* (%)	63 (80.8)	175 (75.1)	0.307
Diabetes mellitus, *n* (%)	24 (31.6)	75 (32.9)	0.832
Hyperlipidemia, *n* (%)	69 (90.8)	191 (83.8)	0.132
Smoking habits: former/current, *n* (%)	17/8 (22.4/10.5)	66/25 (28.9/11.0)	0.505
Alcoholic habits: former/current, *n* (%)	5/5 (6.5/6.5)	22/28 (9.6/12.3)	0.239
Daily aspirin dose			
100 mg, *n* (%)	65 (85.5)	196 (86.0)	0.924
50 mg or 75 mg, *n* (%)	11 (14.5)	32 (14.0)	
Medications taken			
ACEIs/ARBs, *n* (%)	48 (63.2)	114 (50.0)	0.046
Calcium-channel blockers, *n* (%)	24 (31.6)	85 (37.3)	0.369
β-blockers, *n* (%)	53 (69.7)	146 (64.0)	0.365
Nitrates, n (%)	31 (40.8)	68 (29.8)	0.077
Statins, *n* (%)	72 (94.7)	202 (88.6)	0.120
Diuretics, n (%)	11 (14.5)	33 (14.5)	0.999
Proton pump inhibitors, *n* (%)	21 (27.6)	41 (18.0)	0.071

*PCI* Percutaneous coronary interventionm, *TIA* Transient ischemic attacks, *ACEIs* Angiotensin converting enzyme inhibitors, *ARBs* Angiotensin receptor blockers

**Table 2:** Laboratory data of study participants classified by HTPR

Variables	HTPR group (*N* = 76)	non-HTPR group (*N* = 228)	*p* value
Hemoglobin, g/dl	12.6 ± 1.6	13.4 ± 1.6	< 0.001
White blood cell count, ×10^3^/μL	5.9 ± 1.8	6.1 ± 1.5	0.458
Neutrophil percentage, %	61.3 ± 10.9	62.1 ± 9.5	0.507
Platelet count, × 10^3^/uL	174 ± 49	184 ± 51	0.180
Mean platelet volume, fl	8.7 ± 0.8	8.8 ± 1.2	0.334
eGFR, mL/min/1.73 m^2^	65.2 ± 14.7	71.8 ± 17.3	0.002
Serum uric acid, mg/dL	5.83 ± 1.46	5.55 ± 1.27	0.112
Serum creatinine, mg/dL	1.00 (0.86–1.17)	0.98 (0.81–1.12)	0.121
Serum urea, mg/dL	39.97 ± 11.42	40.99 ± 12.92	0.556
Glycosylated hemoglobin, %	5.9 (5.6–6.7)	6.0 (5.6–6.3)	0.905
Triglycerides, mg/dL	94.8 (72.7–130.9)	102.8 (71.8–147.1)	0.436
Total cholesterol, mg/dL	133.0 (113.7–154.2)	132.7 (120.2–152.7)	0.510
HDL-C, mg/dL	41.2 ± 10.8	42.9 ± 10.5	0.243
LDL-C, mg/dL	68.2 (59.5–82.2)	70.0 (59.9–83.1)	0.666
C-reactive protein, mg/L	0.98 (0.32–3.90)	0.73 (0.35–1.70)	0.389

*eGFR* Estimated glomerular filtration rate, *HDL-C* High density lipoprotein cholesterolm, *LDL-C* Low density lipoprotein cholesterol

Linear correlation analysis showed that LTA-AA was significantly correlated with age (*r* = 0.128, *p* = 0.025), hemoglobin (*r* = − 0.216, *p* < 0.001) and eGFR (*r* = − 0.230, *p* < 0.001) for the entire study population. Multivariate regression analysis of HTPR included the following independent variables in the model: age, systolic blood pressure, eGFR, hemoglobin, nitrate use, proton pump inhibitor (PPI) use and ACEI/ARB use. The results showed that eGFR was an independent factor associated with HTPR (OR: 0.984, 95% CI: 0.980–0.988, *p* < 0.001).

### Clinical features of elderly patients with ASCVD classified by renal function

Study participants were stratified by eGFR. Clinical and biologic characteristics with statistical significance are shown in Table 3. In all enrolled patients, 16.1% had a normal eGFR, 52.6% had a slight decrease in eGFR, and 31.3% had a moderate decrease in eGFR. LTA-AA was higher in patients with decreased eGFR than in those with normal eGFR. Patients with moderately decreased eGFR had a higher frequency of HTPR than patients with slightly decreased eGFR or normal eGFR (35.8, 22.5, 12.2%, respectively, *p* < 0.05). Renal function deteriorates with age. As eGFR decreased, a descending trend of diastolic blood pressure levels and hemoglobin levels, as well as an ascending trend of serum creatinine, urea and uric acid levels appeared. Patients with moderately decreased eGFR had higher MPV (mean platelet volume)/PLT (platelet count) ratios and neutrophil percentages compared to those with slightly decreased eGFR (*p* < 0.05). Compared with patients with normal eGFR, patients with moderately decreased eGFR had an increased presence of ischemic stroke/transient ischemic attack, peripheral artery stenosis and hyperlipidemia (*p* < 0.05), and the ratios of ACEIs/ARBs, statins and PPI medications were higher (*p* < 0.05). The results of ROC analysis showed that eGFR levels in all patients were calculated with 70.3 mL/min/1.73m^2^ as a cutoff value to predict HTPR, with an area under the ROC curve of 0.620 (95% CI 0.551–0.689, *p* = 0.002).

**Table 3:** Clinical features of study participants classified by renal function

Variables	G1 group (*N* = 49; eGFR ≥90 mL/min/1.73 m^2^)	G2 group (*N* = 160; eGFR 60–89 mL/min/1.73 m^2^)	G3 group (*N* = 95; eGFR 30–59 mL/min/1.73 m^2^)	*P* value	*P* value		
					G1 vs. G2	G1 vs. G3	G2 vs. G3
eGFR, mL/min/1.73m^2^	94.5 ± 7.3	74.2 ± 8.5	50.8 ± 7.9	< 0.001	< 0.001	< 0.001	< 0.001
HTPR, *n* (%)	6 (12.2)	36 (22.5)	34 (35.8)	0.005	0.117	0.003	0.021
LTA-AA, %	7.75 ± 4.07	9.90 ± 3.83	10.92 ± 4.63	< 0.001	0.002	< 0.001	0.058
Age, years	67 ± 7	76 ± 7	82 ± 5	< 0.001	< 0.001	< 0.001	< 0.001
Diastolic blood pressure, mmHg	77 ± 11	72 ± 10	69 ± 10	< 0.001	0.007	< 0.001	0.025
Hemoglobin, g/dl	14.0 ± 1.6	13.4 ± 1.4	12.4 ± 1.7	< 0.001	0.013	< 0.001	< 0.001
Neutrophil percentage, %	61.1 ± 10.2	59.9 ± 9.4	65.6 ± 9.6	< 0.001	0.458	0.007	< 0.001
PLT, × 10^3^/uL	190 ± 55	186 ± 50	169 ± 47	0.012	0.626	0.016	0.008
MPV, fl	9.4 ± 1.4	8.6 ± 1.1	8.7 ± 1.0	< 0.001	0.004	0.016	0.886
MPV/PLT ratio, fl × 10^−3^uL	0.053 ± 0.018	0.050 ± 0.016	0.056 ± 0.018	0.031	0.248	0.393	0.009
Serum creatinine, mg/dL	0.70 (0.62–0.80)	0.95 (0.85–1.03)	1.21 (1.12–1.32)	< 0.001	< 0.001	< 0.001	< 0.001
Serum urea, mg/dL	31.9 ± 8.0	38.0 ± 9.2	47.0 ± 14.7	< 0.001	0.007	< 0.001	< 0.001
Serum uric acid, mg/dL	5.12 ± 1.32	5.52 ± 1.22	6.04 ± 1.37	< 0.001	0.059	< 0.001	0.002
C-reactive protein, mg/L	0.5 (0.3–1.3)	0.6 (0.3–1.3)	1.4 (0.7–4.1)	< 0.001	0.988	0.006	< 0.001
Medical history							
Ischemic stroke/TIA, *n* (%)	9 (18.4)	48 (30.0)	38 (40.0)	0.026	0.110	0.009	0.102
Peripheral artery stenosis, *n* (%)	0 (0)	19 (11.9)	17 (17.9)	0.007	0.011	0.002	0.182
Hypertension, *n* (%)	33 (67.3)	118 (73.8)	81 (85.3)	0.031	0.432	0.020	0.053
Hyperlipidemia, *n* (%)	35 (71.4)	137 (85.6)	88 (92.6)	0.003	0.023	0.001	0.093
Medications taken							
ACEIs/ARBs, *n* (%)	20 (40.8)	83 (51.9)	59 (61.2)	0.046	0.175	0.015	0.112
Statins, *n* (%)	34 (69.4)	152 (95.0)	88 (92.6)	< 0.001	< 0.001	< 0.001	0.437
Diuretics, n (%)	5 (10.2)	17 (10.6)	22 (23.2)	0.015	0.993	0.059	0.007
Proton pump inhibitors, *n* (%)	2 (4.1)	32 (20.0)	28 (29.5)	0.002	0.008	< 0.001	0.085

*LTA-AA* Light transmission assay with arachidonic acid, *eGFR* Estimated glomerular filtration rate, *PLT* Platelet count, *MPV* Mean platelet volume, *TIA* Transient ischemic attacks, *ACEIs* Angiotensin converting enzyme inhibitors, *ARBs* Angiotensin receptor blockers

## Discussion

Cardiovascular disease and thrombosis are very common complications in patients with renal dysfunction, possibly due to increased platelet activity. In the present study, we reported an association between HTPR and impaired renal function (using the surrogate of eGFR). Our study revealed that decreased eGFR was an independent risk factor of HTPR. HTPR was more common in elderly patients with mildly/moderately decreased eGFR than in those with preserved renal function. Therefore, we suggested that in elderly patients with impaired renal function undergoing aspirin therapy, platelet reactivity should be monitored more intensely to identify HTPR. In clinical practice, the personalized antithrombotic regimen, rather than routine use of aspirin, should be recommended in elderly patients with renal dysfunction.

To date, few studies have been conducted regarding the role of renal function on platelet reactivity. Conflicting results were reached, and most of them were conducted among patients receiving clopidogrel in association with aspirin [14–17]. However, there is evidence of overlap in the antiplatelet effects of aspirin and P2Y12 antagonist [11]. Regarding patients receiving aspirin monotherapy, results from previous studies were also inconsistent. Using the same technology (LTA-AA) in 169 patients with coronary artery disease, Blann et al. found that patients with aspirin resistance had lower eGFR than patients who were sensitive to aspirin [18]. Their data pointed to an association between worsening renal function and aspirin resistance, which was in line with our study. However, Würtz et al. reported that renal function did not correlate with platelet aggregation evaluated by multiple electrode aggregometry or the VerifyNow [19]. These conflicting results may be due to differences in the test methods of platelet reactivity or the inclusion criteria. The underlying mechanism of platelet hyperactivity in patients with impaired renal function might be related to chronic low-grade inflammation, vascular injury [20] and the accumulation of uremic toxins, such as indoxyl sulfate [21] and homocysteine [22]. Furthermore, pre-activation of platelets and platelet turnover [23] also play roles in the pathogenesis of insufficient platelet inhibition by aspirin in patients with impaired renal function.

Besides the impact of renal function, the mechanisms of HTPR are probably multifactorial. Previous studies have specifically addressed the relation between age and HTPR [9, 24–26]. Bobescu et al. reported that low response to aspirin was significantly correlated with age older than 65 years [27]. In our study, all enrolled patients are over 60 years old. There was a positive correlation between age and platelet aggregation rate. Age was a univariate predictor of HTPR, although this dropped out in a multivariate analysis. Future detailed mechanistic research is needed to investigate the relationship of platelet changes with aging and the pathophysiological basis of the antiplatelet response. Elderly patients also represent a category where other factors, such as low body weight, comorbidities or drug combination, have an intrinsic high risk of bleeding [28]. At present, antiplatelet strategies in elderly patients with ASCVD should be cautiously driven by an individualized approach, balancing thrombotic and bleeding risk.

Drug interactions have also been implicated as a potential mechanism of HTPR. PPIs are usually prescribed to patients receiving antiplatelet therapy to decrease the risk of upper gastrointestinal bleeding. Acid suppression with PPIs can increase the potential for mucosal esterases to hydrolyze aspirin to its inactive form. A reduction in gastric absorption results in an increased drug load within the small intestine, where hydrolysis by esterases prior to absorption may reduce bioavailability [29]. This could explain the finding in our study that PPI medication was more common in the HTPR group. Nevertheless, the current guidelines on cardiovascular disease are in favor of PPI prescription in those who are at risk of gastrointestinal bleeding, with the additional benefit that a reduction in dyspepsia may improve adherence [30, 31]. It is worth noting that platelet reactivity should be monitored in these patients.

### Limitations

There were several limitations in this study. First, the present data were observational, with all the inherent limitations of a retrospective analysis. We did not have baseline pretreatment data for platelet function as that would not have been feasible with this particular study design. Prospective or interventional investigations should be conducted in the future. Some clinical and biological parameters were intertwined and inevitable. Larger trials with subgroup analysis and matched-pair analysis are needed. Second, we only used LTA-AA tests as a surrogate measure of aspirin response, rather than specific assessment of its effect on the therapeutic target (i.e., thromboxane B2).

## Conclusions

Among elderly patients receiving antiplatelet therapy with aspirin for secondary prevention of ASCVD, advanced age was correlated with insufficient antiplatelet effects of aspirin. A significant relationship between HTPR and impaired renal function was also observed. Larger trials are needed to assess the clinical impact of this finding and investigate the optimal antithrombotic regimen in elderly patients.

## Data Availability

The datasets used or analysed during the current study are available from the corresponding author on reasonable request.

## Abbreviations

ACEI: Angiotensin converting enzyme inhibitor
ARB: Angiotensin receptor blocker
ASCVD: Atherosclerotic cardiovascular disease
COX: Cyclooxygenase
eGFR: Estimated glomerular filtration rate
HTPR: High on-treatment platelet reactivity
LTA-AA: Light transmission aggregometry with arachidonic acid
MPV: Mean platelet volume
PLT: Platelet count
PPI: Proton pump inhibitor
ROC: Receiver operating characteristic
TXA2: Thromboxane A2
